# The Effect of Age on Gait Speed When Texting

**DOI:** 10.3390/ijerph17020599

**Published:** 2020-01-17

**Authors:** Linson J. Alapatt, Nancye M. Peel, Natasha Reid, Leonard C. Gray, Ruth E. Hubbard

**Affiliations:** 1 Department of Health, Queensland Government, Brisbane, QLD 4000, Australia; LinsonJohn.Alapatt@health.qld.gov.au; 2 Centre for Health Services Research, The University of Queensland, Brisbane, QLD 4102, Australia; n.peel@uq.edu.au (N.M.P.); len.gray@uq.edu.au (L.C.G.); 3 School of Public Health, The University of Queensland, Brisbane, QLD 4006, Australia; n.reid@uq.edu.au

**Keywords:** ageing, gait speed, physical health, dual task test, texting

## Abstract

Texting while walking exerts a high cognitive load, and may be a sensitive test of the integrity of the cognitive–motor interface. We aimed to investigate the association between chronological age and gait speed while texting. A convenience sample of 308 community-dwellers was recruited: n ≥ 50 in each age group (20–29, 30–39, 40–49, 50–59) and n = 100 aged ≥60 years. Gait speed was measured over 10 metres under two experimental conditions: 1) walking at usual pace; 2) walking at usual pace while texting the message “Good morning Harry” on their smartphone. Both median gait speed with and without texting decreased with increasing age (*p* < 0.001). The differences between single- and dual-task gait speed were substantial for each age group and increased after the age of 50 years (*p* < 0.001). Median gait speeds while texting in people aged 50–59 (1.07 m/s) and ≥60 years (1.00 m/s) were below the recommended minimum for safely crossing roads (1.20 m/s). Texting while walking currently exposes people aged 50 and over to considerable environmental hazards. The significant slowing of gait speed while texting from middle age may be a marker of neurodegeneration, a cohort effect, or an appropriate compensatory response to reduce the risk of injury.

## 1. Introduction

Walking has traditionally been viewed as an automatic task requiring little input from higher mental functions. However, an intricate interaction between motor function and cognition is now recognized. Slow gait speed is predictive of multiple adverse outcomes [1] and, although it is also impacted by conditions such as sarcopenia and physical frailty [2], it is strongly associated with prevalent and incident cognitive impairment [3]. Structural and functional brain imaging studies have shown that cognition and motor control share common brain pathways, particularly in the prefrontal and temporal areas [4]. These areas play a crucial role in executive functioning, and seem to be particularly vulnerable to change with “normal ageing” [5,6]. Some age-related changes in gait performance are compensatory and beneficial to maintain stability and reduce the risk of falls, while other changes result in increased risk of injury [5]. Studies investigating dual tasks, which are dependent on intact executive function, reveal that older people are less able to maintain normal ambulation while performing an additional task, particularly talking [7]. Indeed, a review of 11 studies comparing younger and older adults’ performance on various dual-task walking tests found that the dual-task cost was higher for older adults [5].

Recently there has been a proliferation in the use of mobile technology, such that smartphones are now considered an integral part of everyday life. Texting requires the integration of fine motor skills and visual inputs as well as communication planning [8], and thereby exerts a higher cognitive load than talking [9]. Texting while walking may therefore be a more sensitive test of the integrity of the cognitive–motor interface. A recent narrative review of 20 studies, mostly in young healthy adults, concluded that texting was associated with impaired gait performance [10]. Subsequent studies have also shown consistent findings; however, studies continue to be conducted in young adults [11,12,13], or with small sample sizes of as few as 10 participants [12,13,14,15]. Given the increasing use of smartphones and other devices in older age groups [16], the higher dual-task cost of similar activities in older versus younger adults, and the evidence of increasing “pedestrian distraction by technology” [17], there is a need to investigate the impact of texting on walking across age categories. Here, we aimed to investigate the association between chronological age and gait speed while texting and not texting.

## 2. Materials and Methods 

### 2.1. Study Design and Setting

A comparative study measured the gait speed of pedestrians while walking in a public outdoor space on a level surface. The chosen setting was the Eleanor Schonell Bridge, which spans 390 metres over the Brisbane River in Queensland, Australia and is separated into distinct areas for pedestrian, bicycle and vehicular traffic (buses only). A 10-m distance was marked with tape in the centre of the pedestrian walkway of the bridge and there was a clear view for the participant from either end. 

### 2.2. Participants

Convenience sampling was used to recruit 308 participants from the community dwelling population aged 20 and over: at least 50 each in the age groups of 20–29 years, 30–39 years, 40–49 years, 50–59 years, and 100 participants aged 60 years and older. Participants were excluded if they were younger than 20 years of age, did not use a smart phone or if they had any disability or conditions that affected their ability to walk 10 m. Recruitment took place over the period June to August 2017 during the hours between 2:00 and 4:30 pm. 

### 2.3. Measures

Gait speed was measured under two experimental conditions: 1) walking at usual pace; 2) walking at usual pace while texting the message “Good morning Harry” on their smartphone. The spelling accuracy and completion of the message were noted. Autocorrect was turned off to identify typing errors. No instructions were given to participants regarding the texting accuracy, and they were free to correct their errors. Errors in the text messages were recorded if they were inaccurate or not completed. In each condition, participants walked in a straight line for 10 m. A standing start was used, and the participants were timed using a stopwatch from the time of the instruction to start to the time of the first foot fall across the finish line. Gait speed was calculated by dividing the distance by the time taken, in metres per second (m/s). This study took place on a smooth surface with no obstacles. The investigator walked alongside each participant during the procedure to prevent any falls. 

### 2.4. Ethics 

All procedures were approved by the University of Queensland Human Research Ethics Committee and conformed to the declaration of Helsinki. Each participant provided written informed consent. Because the site chosen was a public place (pedestrian walkway on a bridge crossing the Brisbane River), “gatekeeper” approval to use the bridge for the study was sought from the Brisbane City Council, who also sought the approval of the Bus Drivers’ Union for assurance that the activity would not distract drivers. 

### 2.5. Statistical Analysis

Frequency distributions were used to describe the characteristics of the sample population by age group and gender, with differences in proportions examined using chi-square tests or Fisher’s exact test if cell numbers were less than five. Depending on the distribution of the data, walking times (with and without texting) across age groups were compared using parametric (comparison of means) or non-parametric (comparison of medians) tests. Multiple pairwise comparisons between age groups were carried out using Dunn–Bonferroni post hoc tests. Differences between walking times under the two conditions were compared using tests for paired data. Data were analysed using SPSS, Version 25 (IBM Statistics, Armonk, NY, USA). 

## 3. Results

### 3.1. Difference in Gait Speed 

Of the 308 participants, there were 135 females (43.8%), with no significant sex differences across age groups (Table 1). Under each of the two conditions, median gait speed (GS) was significantly different by age group (*p* < 0.001) but not by gender (*p* = 0.97 for single-task GS; *p* = 0.72 for dual-task GS). Gait speed (median [interquartile range]) while texting (1.16 [1.01, 1.34] m/s) was significantly slower than when not texting (1.47 [1.40, 1.58] m/s) (*p* < 0.001). 

Pairwise comparisons between age groups showed that, for usual gait speed, the ≥60 age group had significantly lower gait speed than those aged 50–59 (*p* = 0.040), 40–49 (*p* < 0.001), 30–39 (*p* = 0.004) and 20–29 (*p* < 0.001). Gait speed while texting was also significantly slower in the ≥60 age group compared to the other age groups: 50–59 (*p* = 0.003), 40–49 (*p* < 0.001), 30–39 (*p* < 0.001) and 20–29 (*p* < 0.001). In addition, those aged 50–59 had significantly slower gait speed while texting than the younger age groups (*p* < 0.001). Table 2 shows the difference in gait speeds by age group.

**Figure 1:**
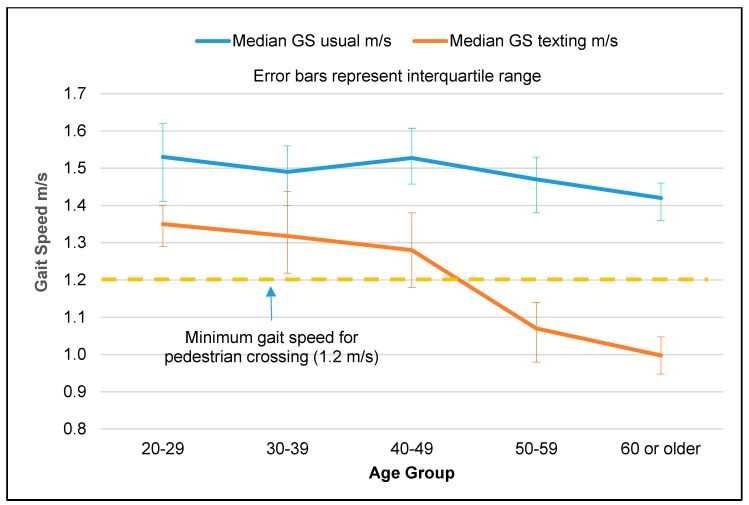
Comparison of gait speed (usual and while texting) by age group.

The difference between the two gait speed measures across age groups was significant (*p* < 0.001) (Table 2), and pairwise comparisons showed that this difference was significantly higher in the age groups ≥50 compared to younger age groups (*p* < 0.001), as shown in Figure 1. Comparing the 50–59 and ≥60 age groups, the difference between the two gait speed measures was not significant.

### 3.2. Difference in Texting Errors

During the texting conditions, the proportion of participants with incomplete or incorrect texts differed across age groups (Table 1). All participants younger than 30 years of age completed the text correctly, while 7.7% (n = 4) of those 30–39 years of age and 12.7% (n = 7) aged between 40–49 years had incomplete or incorrect text. The proportion of errors increased exponentially above the age of 50, to 62% (n = 32) for those aged 50–59 years and 81% (n = 81) for those 60 years and older.

## 4. Discussion

In this study, we examined the effect of age on walking speed while texting on a smartphone. Dual-tasking reduced gait speed across all age groups, but with a significant decline after 49 years. Similarly, the proportion of incorrect or incomplete texts increased with age and rose exponentially in the 50–59 and 60-plus age groups. One explanation for our findings is that texting-while-walking may be a sensitive test of age-associated neurodegenerative disease, since it has been found that some domains of cognitive function (such as reasoning and verbal fluency) show evidence of decline from middle age (45–49 years) [6,18]. 

An additional finding in this study was that median gait speeds while texting in people aged 50–59 (1.07 m/s) and ≥60 years (1.00 m/s) were below the recommended minimum for safely crossing roads (1.20 m/s) [19]. This is consistent with previous literature, where simulator studies have shown the detrimental effects of dual-tasking on street crossing performance. These studies found that the need for decision making and planning was greater in texting while walking scenarios, thus requiring participants to physically divert their attention from the crossing scene [20]. Another simulator study, assessing dual-task performance in a virtual reality environment where participants crossed a simulated street by walking on a treadmill, found that older adults at risk of falls experienced more collisions with oncoming cars and had longer street crossing times [21]. Other studies have shown that dual tasking slows gait speed and texting accuracy in young healthy individuals [22,23], yet pace remains within normal limits [23]. Nevertheless, pedestrian injuries related to mobile-phone use are highest for people under 31 years of age [24], which could suggest a compensatory mechanism in older people, or a higher frequency of device use in younger adults and teenagers [24]. Regardless, this has both personal safety and public health implications. Texting lanes and other environmental modifications (e.g., pedestrian crossing lights projected onto the ground) have been introduced in some provinces in China and Germany to reduce the pedestrian risks for “distracted walkers” [25]. 

A strength of our study is that the sample size was moderately large, and covered age ranges from 20 to 60 years and older. Data collection was done by a single investigator, at one site, using a consistent methodology. Participants were familiar with texting and used their own smart-phones so as to eliminate the possibility of having to learn a new operating system.

We also acknowledge limitations. This was a cross-sectional study with convenience sampling. The duration of smartphone use (number of years using the device) and intensity of usual texting activity (number of hours per day spent texting) were not recorded, and may have been factors influencing gait speed while texting, especially in the older age groups who may not be “digital natives” [23]. Another limitation was a lack of data collection regarding age-related factors such as participants’ educational attainment or current cognitive status. Furthermore, we did not capture several important factors known to affect gait speed, such as body mass index, type of footwear and other physical limitations. Future studies may benefit from comparing a dual task with texting to other activities, such as talking or numerical activities.

## 5. Conclusions

In this study, increased cognitive load was associated with slower gait speed, particularly for people aged more than 49 years. This, in conjunction with simulator studies showing that older adults under cognitive load are more likely to make judgement errors on street crossings and the increasing use of technology in older age groups, presents an emerging but significant public health issue. Behavioural strategies such as those leading to “texting lanes” in other countries may be a useful tool to address this issue more broadly [26]. Targeted approaches could include specific training to improve dual-tasking in both younger and older people [27,28], with evidence that combined physical and cognitive training can lead to cognitive improvement in attention and executive function [29]. While neurodegeneration is not the only factor contributing to the slowing of gait speed, practice in texting while walking therefore has the potential to maintain the complex pathways at the cognitive–motor interface.

## Figures and Tables

**Table 1:** Characteristics of the study population by age group.

Measure	Age Group ^a^					Total 308
	20–29 Years 50 (16.2)	30–39 Years 52 (16.9)	40–49 Years 55 (17.9)	50–59 Years 51 (16.6)	≥60 Years 100 (32.5)	
Females ^a^	18 (36.0)	26 (50.0)	24 (43.6)	23 (45.1)	44 (44.0)	135 (43.8)
Usual GS ^b^	1.53 (1.42, 1.63)	1.49 (1.41, 1.57)	1.53 (1.46, 1.61)	1.47 (1.40, 1.55)	1.42 (1.37, 1.47)	1.47 *** (1.39, 1.58)
Texting GS ^b^	1.35 (1.30, 1.41)	1.32 (1.22, 1.44)	1.28 (1.17, 1.37)	1.07 (1.01, 1.17)	1.00 (0.95, 1.05)	1.16 *** (1.01, 1.34)
Text errors ^a^	0 (0)	4 (7.7)	7 (12.7)	32 (62.7)	81 (81.0)	124 (40.3) ***

GS: gait speed in m/s; ^a^ *n* (%); ^b^ Median (inter-quartile range); *** *p* < 0.001 (distribution significantly different across age groups).

**Table 2:** Difference in gait speed by age group.

Age Group	*n* (%)	Usual GS ^a^	Texting GS ^a^	Absolute Difference ^a^	% Decrease in GS ^a^
20–29	50 (16.2)	1.53	1.35	0.16	10.74
30–39	52 (16.9)	1.49	1.32	0.17	11.08
40–49	55 (17.9)	1.53	1.28	0.25	16.71
50–59	51(16.6)	1.47	1.07	0.38	25.93
≥60	100 (32.5)	1.42	1.00	0.43	30.37
Total	308 (100)	1.47 ***	1.16 ***	0.32 ***	22.25 ***

GS: gait speed in m/s; ^a^ Median; *** *p* < 0.001 (distribution significantly different across age groups).
